# A Time-Resolved Fluorescent Lateral Flow Immunoassay for the Rapid and Ultra-Sensitive Detection of AFB_1_ in Peanuts and Maize

**DOI:** 10.3390/foods14244218

**Published:** 2025-12-09

**Authors:** Yunrui Xing, Suzhen Yang, Lu Fan, Xiaofei Hu, Shengnan Liu, Yao Wang, Yaning Sun

**Affiliations:** 1 Institute for Animal Health, Henan Academy of Agricultural Sciences, Zhengzhou 450002, China; xyrui1201@163.com (Y.X.); yang-suzhen@163.com (S.Y.); fanlu.lab@gmail.com (L.F.); huxf1972@126.com (X.H.); 2 Technology Center of Zhengzhou Customs, Zhengzhou 450003, China; amylsn_2008@126.com; 3 Henan International Joint Laboratory of Food Green Processing and Quality Safety Control, College of Food and Bioengineering, Henan University of Science and Technology, Luoyang 471023, China; wangyao@haust.edu.cn; 4 Longhu Laboratory of Advanced Immunology, Zhengzhou 450046, China

**Keywords:** lateral flow immunoassay, aflatoxin B_1_, time-resolved fluorescence microspheres, on-site rapid detection

## Abstract

Aflatoxin B_1_ (AFB_1_), a highly toxic and carcinogenic mycotoxin, poses significant public health risks due to its widespread contamination of staple food crops such as peanuts and maize. Although conventional lateral flow immunoassays (LFIAs) are widely employed for rapid on-site screening, their limited sensitivity frequently compromises accurate quantification at trace levels. To improve the analytical performance of LFIAs, we developed a novel time-resolved fluorescence-based lateral flow immunoassay (TRFN-LFIA) by integrating reverse artificial antigen labeling with time-resolved fluorescence signal amplification. This method enhances detection sensitivity and enables rapid, ultra-sensitive, visible, and quantitative determination of AFB_1_ in peanut and maize samples. Under optimized conditions, the TRFN-LFIA achieved a visible limit of detection (vLOD) of 0.30 ng/mL (2.22 µg/kg), a quantitative limit of detection (qLOD) of 0.04 ng/mL (0.30 μg/kg), and a half-maximal inhibitory concentration (IC_50_) of 0.09 ng/mL. Recoveries from spiked peanut and maize samples ranged from 81.33% to 117.86%, with coefficients of variation (CVs) below 13.04%. Analysis of 21 real samples (13 maize and 8 peanut samples) yielded results highly consistent with those obtained by liquid chromatography–tandem mass spectrometry (LC-MS/MS). Moreover, the method demonstrates significant advantages in terms of detection speed, cost-effectiveness, and operational convenience. Therefore, the results established the TRFN-LFIA method as a reliable and practical tool for on-site rapid detection of AFB_1_ in contaminated food matrices, providing both a rapid and accurate approach for trace-level quantification and a novel strategy for enhancing the sensitivity of lateral flow immunoassays.

## 1. Introduction

Aflatoxin (AFT) is a toxic secondary metabolite predominantly produced by filamentous fungal species, particularly Aspergillus flavus and Aspergillus parasiticus [1]. At present, 21 aflatoxin variants have been identified, with the most commonly occurring being aflatoxins B_1_ (AFB_1_), B_2_ (AFB_2_), G_1_ (AFG_1_), and G_2_ (AFG_2_), as well as their metabolic derivatives M_1_ (AFM_1_) and M_2_ (AFM_2_) [2,3]. Among these variants, AFB_1_ is particularly concerning due to its high toxicity and carcinogenicity [4]. It is widely present in nature, and a variety of plant and animal foods may be contaminated, such as cereals, oilseeds, nuts, fruits and their products [5,6,7,8].

AFB_1_ can be highly toxic to animals and humans, depending on the dose, exposure duration, and affected organs [9,10]. Studies have shown that AFB_1_ is closely related to various toxic effects, including hepatotoxicity [11,12,13], enterotoxicity [14,15], neurotoxicity [16,17], nephrotoxicity [18], immunotoxicity [19,20], and genotoxicity [21]. AFB_1_ contamination represents a critical global public health and food safety concern and has been classified as a Group 1 carcinogen by the International Agency for Research on Cancer (IARC) of the World Health Organization (WHO) [22,23]. Given its widespread occurrence and high toxicity, many countries have developed specific maximum permitted levels (MPLs) for AFB_1_ in food and animal feed [24]. For example, China has established MPLs for AFB_1_ in food and feeds at 0.5–20 µg/kg and 10–50 µg/kg, respectively [25,26]. The European Union has set MPLs of AFB_1_ at 2 µg/kg in cereals and within the range of 5 to 20 µg/kg for feed materials and compound feeds [27,28]. In India, MPLs for AFB_1_ were set between 1 and 20 µg/kg for food products and from 0 to 50 µg/kg for animal feed, respectively [29].

Given the health risks, rapid, sensitive, and on-site detection methods are critical for controlling AFB_1_ contamination [30]. Currently, methods for detecting AFB_1_ primarily include immunoassay techniques and instrumental analysis [28,31]. Among these methods, the lateral flow immunoassay (LFIA) offers sensitivity, specificity, and simplicity without the need for complex instruments or technicians. It can also be used for real-time detection [32]. Colloidal gold is the most widely used labeling material in LFIAs; however, it is limited by low sensitivity, with a vLOD typically exceeding 1 ng/mL. Given the simplicity and convenience of the strip assay method for users, significant efforts have been devoted in recent years to improving its sensitivity. The main strategies for enhancing the detection sensitivity of LFIAs involve selecting novel labeling materials, precisely regulating antibody concentrations, and extending the reaction time between the target antigen and antibody. TRFN-LFIA is an immunochromatographic assay that uses time-resolved fluorescence microspheres (TRFM) as labels. TRFM exhibits a significantly large Stokes shift exceeding 150 nm and has a fluorescence lifetime that is 5 to 6 orders of magnitude greater than that of background signals [33]. Therefore, by delaying the detection time, interference from various non-specific fluorescence can be effectively eliminated; this approach has been demonstrated to markedly improve detection sensitivity compared to gold-based strip assays [34]. The traditional TRFN-LFIA method for detecting AFB_1_ involves labeling an AFB_1_ monoclonal antibody (mAb) with TRFM while using artificial antigen as a test line (T-line) [35]. However, labeling antibodies does not allow accurate control over both their quantity and labeling sites due to steric hindrance which affects detection sensitivity. To address this challenge in this study, TRFM was used to label artificial antigens while adding antibodies in free form into samples. This new TRFN-LFIA method was established to detect AFB_1_ residues in food matrices, achieving further improvements in immunochromatography technology’s sensitivity.

## 2. Material and Methods

### 2.1. Reagents and Equipment

Zearalenone (ZEN) and ochratoxin A (OTA) were sourced from Sigma (Burlington, MA, USA). AFB_1_, AFB_2_, AFM_1_, AFG_1_, AFG_2_, T2, and Deoxynivalenol (DON) were obtained from Aladdin (Shanghai, China). TRFM (MF02, -COOH, 10 mg/mL, 200 nm) was purchased from Made New (Changsha, China). 1-Ethyl-3-(3-dimethylaminopropyl) carbodiimide hydrochloride (EDC·HCl) was acquired from Bulk Chem (Quzhou, China). Sulf-N-hydroxysuccinimide (NHS) was purchased from Thermo (Waltham, MA, USA). Bovine serum albumin (BSA, IgG-free/protease-free) was purchased from Jackson (West Grove, PA, USA). Tween-20 was purchased from Merck & Co. Inc. (Kenilworth, NJ, USA). Staphylococcal protein A (SPA), Chicken IgY, and Goat anti-chicken IgY (IgG) were purchased from Seebio (Shanghai, China).

AFB_1_-BSA and Anti-AFB_1_ mAb were prepared previously by our laboratory, and the mAb exhibited an IC_50_ value for AFB1 of 0.05 ng/mL. Absorbent pads, nitrocellulose membrane (NC, HF13502S25, 30 × 2 cm^2^) and fiberglass came from Millipore (Bedford, MA, USA). All other chemical reagents are analytical purity.

The CM 4000 cutter, XYZ Biostrip dispensing platform, and TSR3000 strip reader were acquired from Bio-Dot (Richmond, CA, USA). The ultrapure water purification system was obtained from Millipore (Bedford, MA, USA). A Zetasizer Nano ZS90 was purchased from Malvern Instruments (Worcestershire, UK). The immunofluorescence card reader (FIC-Q1; 365 nm/610 nm TRFN) was sourced from Helmen (Suzhou, China).

### 2.2. Preparation of TRFM Probes

**Figure 1:**
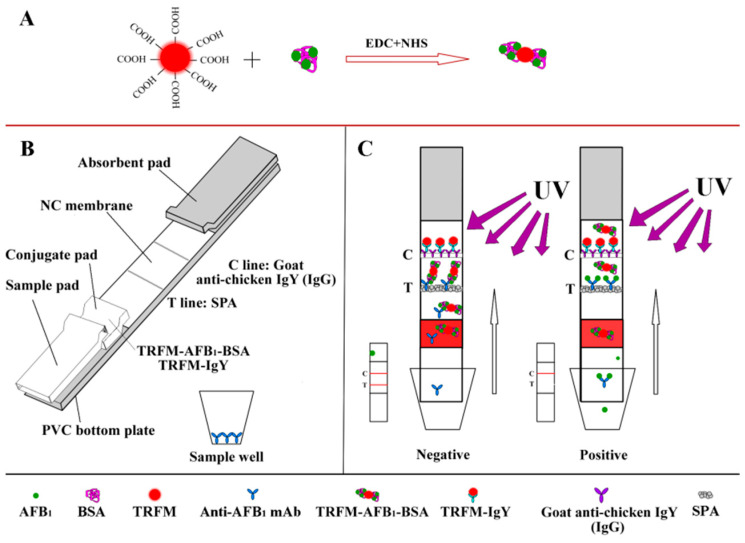
Schematic of the TRFN-LFIA. (**A**) Preparation of the TRFM probes; (**B**) Basic structure of the TRFN-LFIA; (**C**) Schematic of the TRFN-LFIA reaction principle.

To fabricate the TRFM probes, AFB_1_-BSA conjugates were conjugated with TRFM particles as illustrated in Figure 1A. Briefly, 2 mg of TRFM was dispersed into 1.8 mL of double-distilled water (DDW), followed by the sequential addition of EDC·HCl and NHS solutions, each diluted to a concentration of 2 mg/mL with DDW. The volume ratio of EDC·HCl to NHS was 1:1. The reaction mixture was stirred at room temperature for 30 min, followed by centrifugation at 12,000 rpm for 15 min. The supernatant was carefully aspirated, and the resulting pellet was resuspended in 2 mL of borate buffer (10 mmol/L, pH 8.0) containing 0.6 mg of AFB_1_-BSA, followed by incubation under gentle stirring at room temperature for 3 h. Subsequently, 8 mg of casein was slowly added to the solution. The reaction mixture was stirred for 1 h at room temperature and then centrifuged at 12,000 rpm for 15 min. After removal of the supernatant, the pellet was resuspended in 2 mL of suspension buffer (20 mmol/L borate buffer, pH 8.5, supplemented with 1% BSA, 3% trehalose, and 0.03% sodium azide). The synthesis of TRFM-chicken IgY probes was performed using the same procedure [36].

### 2.3. Preparation of the TRFN-LFIA

The test strip components are illustrated in Figure 1B. The conjugate pad was coated with TRFM-AFB1-BSA and TRFM-labeled chicken IgY at a volume of 8 µL/cm. The SPA (which binds to the Fc region of antibodies) and goat anti-chicken IgY (IgG) were immobilized on the NC membrane as the test line (T-line) and control line (C-line), respectively. The anti-AFB_1_ mAb was dissolved in a stabilization buffer containing 10 mM PBS (pH 7.4), 1% BSA, 10% trehalose, 0.5% Tween-20, and 0.2% Proclin 300. The diluted mAb was then dispensed into sample wells at a dosage of 2.4 ng per well, followed by freeze-drying in a lyophilizer for 24 h. The sample wells were then sealed and stored at room temperature in a package containing a desiccant. Subsequently, the NC membrane, sample pad, conjugate pad, and absorbent pad were sequentially assembled onto a PVC backing plate with overlaps of 1–2 mm before being cut into strips of width 3 mm. Finally, the strips were hermetically sealed in a packaging bag with desiccant and stored at room temperature until use.

### 2.4. Detection Principle of the TRFN-LFIA

When a sample is added to the sample well, the anti-AFB_1_ mAb is dissolved in the sample (Figure 1C). AFB_1_ in the sample binds specifically to the mAb, forming an antigen–antibody complex. This complex migrates through the conjugate pad without interacting with TRFM-labeled AFB_1_-BSA and proceeds to the test line (T-line), where it is captured by protein A (SPA). Because the antigen–antibody complex does not carry TRFM, there is no fluorescence signal at the T-line. The competition between sample AFB_1_ and TRFM-AFB_1_-BSA for anti-AFB_1_ mAb binding sites results in an inverse relationship between T-line fluorescence intensity and AFB_1_ concentration. Goat anti-chicken IgY (IgG) was immobilized on the C-line, and independent TRFM-chicken IgY was specially prepared for this purpose. Finally, an immunofluorescence card reader was used to measure T-line fluorescence intensity.

### 2.5. Sample Preparation

The cereal samples were crushed and filtered through an 80-mesh. Subsequently, 5 g of each sample was extracted with 10 mL of 70% methanol [28]. The extraction mixture was shaken vigorously for 15 min and then centrifuged at 4000 rpm for 5 min. The supernatant was collected and diluted at least two-fold with 0.1 mol/L PBS (pH 7.4) for analysis.

### 2.6. Assay Procedure

The sample wells were pre-coated with anti-AFB_1_ mAb. A volume of 100 μL of standard or sample solution was added to each well and mixed thoroughly to ensure complete dissolution of the anti-AFB_1_ mAb. Following a 5 min incubation, the test strips were inserted into the wells. After 15 min of reaction, semi-quantitative results were obtained by visual inspection under UV light, while quantitative results were determined using an immunofluorescence card reader.

### 2.7. Evaluation of Sensitivity

To evaluate the sensitivity of the TRFN-LFIA, a series of AFB_1_ concentrations (0, 0.025, 0.05, 0.075, 0.125, 0.2, 0.3 ng/mL) were prepared by serial dilution in sample buffer solution. The assay was performed according to the established procedure using the TRFN-LFIA and an immunofluorescence card reader. For semi-quantitative analysis, the test strips were visually inspected under UV light, and the visible limit of detection (vLOD) was defined as the lowest concentration at which no fluorescence signal was observed at the T-line [37]. For quantitative analysis, each spiked level was measured in triplicate using the immunofluorescence card reader to generate standard curves, determine IC_50_ values, establish linear ranges, and calculate quantitative limits of detection (qLOD). Standard curves were constructed by plotting B/B_0_ values against the logarithm of AFB_1_ concentration, where B and B_0_ represent the T-line signal intensities in the presence and absence of competitive antigen, respectively [38]. Assay sensitivity was assessed based on the IC_50_ values derived from the standard curves. The linear range was defined as the concentration interval yielding 20–80% inhibition of B/B_0_ [35]. The quantitative LOD (qLOD) was determined as the analyte concentration corresponding to 80% of the B/B_0_ value on the standard curve [37].

### 2.8. Evaluation of Specificity

Specificity, expressed as cross-reactivity (CR, %), was determined by assessing reactivity with other mycotoxins, including AFB_2_, AFM_1_, AFG_1_, AFG_2_, ZEN, OTA, T2, and DON. CR (%) was expressed as the percentage of the IC_50_ value of the target analyte to analogues [37].

### 2.9. Evaluation of Accuracy and Precision

LC–MS/MS was employed as a reference analytical method to confirm AFB1 negative status in peanut and maize samples. The accuracy and precision of the methods, represented by recovery and coefficient of variation (CV), were determined through fortification with AFB_1_ at four concentrations (1, 2, 5, and 25 µg/kg). The spiked samples were analyzed using TRFN-LFIA, and the concentration of AFB_1_ in the extract solutions was calculated based on the standard curve. The mass content of AFB_1_ in samples was calculated based on a formula.

$$C1=\frac{C2\times V\times \mathrm{DF}}{M}\times EC$$

C1: The mass concentration of AFB_1_ in grains.

C2: The volume concentration of AFB_1_ in the extract solution.

V: Volume of extract solution.

DF: The dilution factor of the extract solution.

EC: Extraction coefficient.

### 2.10. Analysis of Naturally Contaminated Samples

Twenty-one naturally contaminated samples (13 maize and 8 peanuts) were simultaneously analyzed by the TRFN-LFIA and LC-MS/MS, and each sample was detected in triplicate. Comparisons were conducted using linear regression analysis, with the regression lines constrained to a zero intercept. The resultant correlation coefficients (R^2^) and slopes were calculated to determine assay variability and responsiveness, respectively, between the test strips and LC-MS/MS [39].

### 2.11. Comparison and Analysis of Different Detection Modes

The sensitivity of immunological detection methods is primarily determined by the performance of core raw materials, such as antigens and antibodies. In this study, using the same antibodies and artificial antigens, AFB_1_ immunochromatographic assays were constructed based on the conventional detection mode through systematic optimization of experimental conditions. These assays employed colloidal gold and time-resolved fluorescent microspheres as labeling materials. Their sensitivities were subsequently evaluated and compared with that of the newly developed TRFN-LFIA.

## 3. Results

### 3.1. Optimization of TRFN-LFIA

Taking the EDC·HCl and NHS amount, AFB_1_-BSA labeling amount, anti-AFB_1_ mAb amount, the TRFM probe amount, SPA content in the T-line, strip reaction time as factors, we designed the test to fulfill the optimal test parameters of the strip. All quantitative figures of merit (i.e., linear dynamic range, LOD, accuracy, precision, sensitivity, and specificity) were calculated based on the values obtained from the card reader.

#### 3.1.1. Optimization of the Labeling of Probe

**Figure 2:**
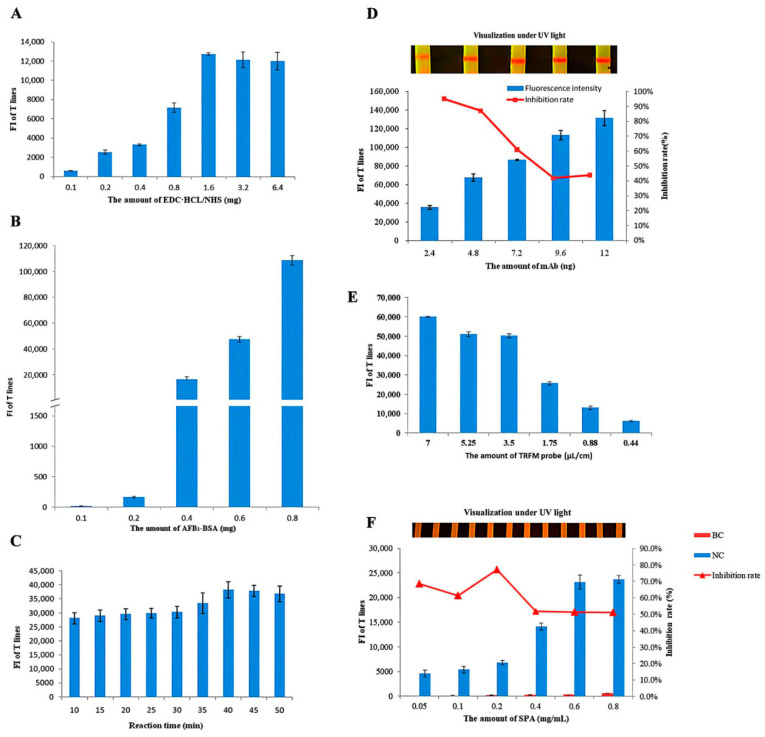
Optimization of TRFN-LFIA. (**A**) The optimization of EDC·HCL and NHS amount; (**B**) The optimization of AFB_1_-BSA; (**C**) The optimization of TRFN-LFIA reaction time amount; (**D**) The optimization of anti-AFB_1_ mAb amount; (**E**) The optimization of TRFM probe amount; (**F**) The optimization of SPA concentration.

To improve the labeling efficiency of AFB_1_-BSA, the amounts of EDC·HCL, NHS, and AFB_1_-BSA were optimized. TRFM and AFB_1_-BSA were added in amounts of 2 mg and 0.3 mg, respectively, with a volume ratio of 1:1 between EDC·HCL and NHS. Figure 2A shows the optimization of EDC·HCL and NHS as well as the change in fluorescence with increasing amounts of each. When the amounts of EDC·HCL and NHS reached 1.6 mg, the reaction reached saturation, and the fluorescence signal intensity achieved its maximum value. Furthermore, as the amount increased, the fluorescence signal reached a plateau.

The results (Figure 2B) indicated that the concentration of AFB_1_-BSA had a significant impact on the fluorescence signal intensity. As the amount of AFB_1_-BSA increased, the fluorescence intensity gradually rose, and the differences among the groups were obvious, suggesting that the input amount was a key factor in regulating the fluorescence intensity. When the amount exceeded 0.4 mg, the fluorescence signal met the detection requirements; considering the signal intensity and reagent cost comprehensively, 0.6 mg was selected as the optimal amount for subsequent experiments.

#### 3.1.2. Optimization of the TRFN-LFIA Reaction Time

As shown in Figure 2C, extending the reaction time can enhance fluorescence intensity to a certain extent; however, excessively prolonged durations lead to reduced detection timeliness. An appropriate reaction time is crucial for achieving an optimal balance between detection accuracy and operational efficiency. When the reaction time is less than 10 min, the fluorescence signal is too weak to meet sensitivity requirements; conversely, when it exceeds 30 min, the time-saving advantage inherent in rapid detection methods is compromised. Comprehensive analysis demonstrates that the optimal reaction time range is 20–30 min. Within this interval, the fluorescence signal remains stable, allowing sufficient time for reliable signal acquisition while preserving the method’s efficiency. Therefore, a reaction time of 20–30 min was adopted for all subsequent experiments.

#### 3.1.3. Optimization of Anti-AFB_1_ mAb Amount

The amount of anti-AFB_1_ mAb used affects the fluorescence intensity and sensitivity of the test strip. If the amount of anti-AFB_1_ mAb is too low, the fluorescence signal might be too weak, whereas an excessively high amount might hinder sensitivity. The amount of anti-AFB_1_ mAb added to each well was determined by the fluorescence intensity of AFB_1_ in negative samples and the inhibition rate of AFB_1_ on the strip at 0.25 ng. Figure 2D shows that fluorescence intensity increased with increasing amounts of anti-AFB_1_ mAb but also with decreasing inhibition rates. T-line visualization under UV light showed that the fluorescence signal was sufficient at 2.4 ng; thus, the optimal amount of anti-AFB_1_ mAb was selected to be 2.4 ng per well.

#### 3.1.4. Optimization of TRFM Probe Amount

The amount of TRFM probe significantly influences fluorescence intensity. In the assay, AFB_1_ in the sample first binds to the antibody, after which the unbound antibody interacts with the fluorescent probe and is subsequently captured by SPA immobilized on the test line, resulting in signal generation. As shown in Figure 2E, fluorescence intensity increased with higher amounts of TRFM probe. However, excessive probe loading led to elevated nonspecific binding and background staining on the NC membrane. A concentration of 1.75 μL/cm yielded minimal background interference while maintaining strong signal intensity. Therefore, 1.75 μL/cm was selected as the optimal application volume for the TRFM probe in subsequent experiments.

#### 3.1.5. Optimization of the Concentration of SPA

SPA was immobilized on the NC membrane at concentrations of 0.05, 0.1, 0.2, 0.4, 0.6, and 0.8 mg/mL for strip assembly. The optimal SPA concentration was determined based on the intensity of the T-line observed in both blank and negative control samples, as well as the inhibition rates of the test strips. Results show that increasing SPA concentration led to a progressive increase in both T-line signal intensity and inhibition rate (Figure 2F). At 0.4 mg/mL, SPA yielded a strong and clearly visible fluorescence signal with minimal background interference. Furthermore, this concentration achieved an excellent inhibition rate. Therefore, 0.4 mg/mL was selected as the optimal concentration for SPA in subsequent experiments.

### 3.2. Sensitivity

**Figure 3:**
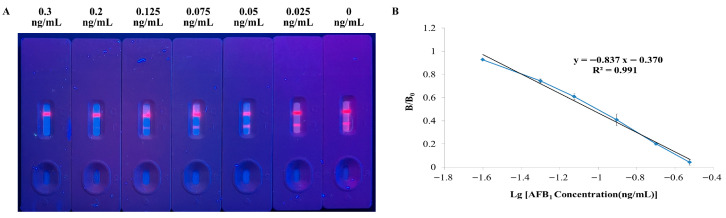
Sensitivity and standard curves. (**A**) Visible detection sensitivity of TRFN-LFIA; (**B**) Standard curve for TRFN-LFIA.

The sensitivity of the TRFN-LFIA was evaluated by detecting a series of AFB_1_ solutions. The vLOD of the TRFN-LFIA was 0.30 ng/mL (Figure 3A). The IC_50_ value, obtained from the standard curves (Figure 3B), was 0.09 ng/mL, which is close to the detection limit of 0.05 ng/mL for the ELISA method. The linear range was 0.04–0.21 ng/mL, and the qLOD was 0.04 ng/mL. This establishes that the TRFN-LFIA has a lower LOD compared to a gold-based LFIA (0.77 µg/kg, 0.25 ng/mL, and 0.59 µg/kg) [37,40,41].

Semi-quantitative testing through visualization enables rapid on-site screening without specialized equipment, whereas quantitative testing offers precise numerical results. Both methods possess distinct advantages and are applicable to different scenarios.

### 3.3. Specificity

**Figure 4:**
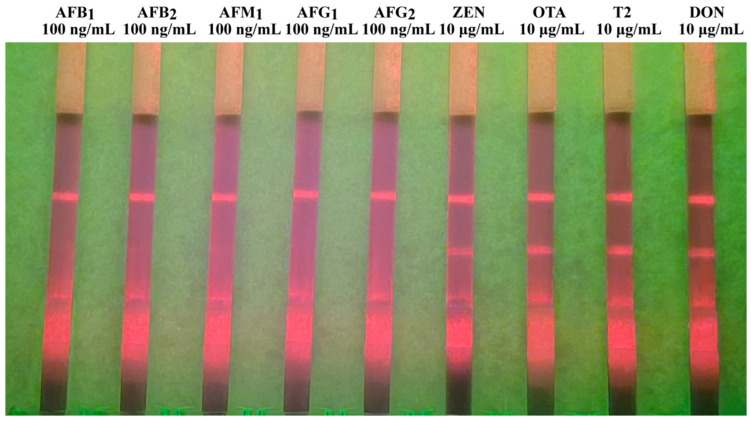
The specificity of TRFN-LFIA.

Structural and functional analogs of AFB_1_ were used to verify the specificity of TRFN-LFIA. The results are shown in Figure 4. TRFN-LFIA recognized only structurally similar analogues and TRFN-LFIA exhibited low cross-reactivity (CR < 5.971%) for four other structurally and functionally similar compounds (Table 1). These results demonstrate good specificity of TRFN-LFIA.

### 3.4. Accuracy and Precision

AFB_1_ standard solutions were added to the negative peanut and maize samples, respectively, to achieve concentrations of 1, 2, 5, and 25 µg/kg. Subsequently, the samples were analyzed using TRFN-LFIA, and the results are presented in Table 2. The recovery rates of AFB_1_ in peanuts and maize ranged from 86.91% to 108.97% and from 81.33% to 117.86%, with corresponding CVs ranging from 6.99% to 13.29% and from 4.36% to 13.04%, respectively. These results indicate good accuracy of TRFN-LFIA, which is suitable for the rapid detection of AFB_1_ in peanuts and maize.

### 3.5. Analysis Results of Naturally Contaminated Samples

**Figure 5:**
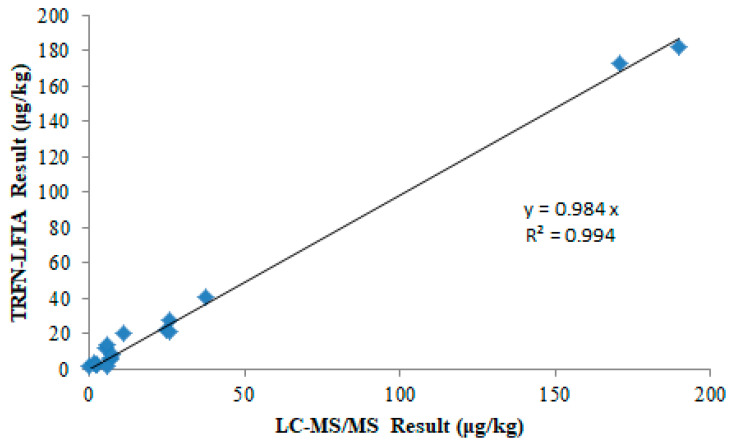
Correlation between TRFN-LFIA and LC-MS/MS measurements of AFB_1_ in naturally contaminated samples.

Twenty-one natural samples (13 maize and 8 peanut samples) were analyzed to validate the practical applicability of TRFN-LFIA, with LC-MS/MS serving as the reference method. TRFN-LFIA screening revealed AFB_1_ levels below the detection limit in four samples, while LC-MS/MS identified five samples as negative for AFB_1_. A linear regression analysis was performed by placing the concentrations measured by LC-MS/MS on the x-axis and those measured by TRFN-LFIA on the y-axis, as illustrated in Figure 5. The regression analysis yielded an R^2^ value of 0.994, reflecting strong concordance between the two methodologies. Furthermore, the calculated slope of 0.984 suggests TRFN-LFIA measurements exhibited marginally reduced values, compared to LC-MS/MS results. These findings collectively validate TRFN-LFIA as a reliable analytical technique for the quantitative determination of AFB_1_ contamination food matrices.

### 3.6. Comparison Results of Different Detection Modes

**Figure 6:**
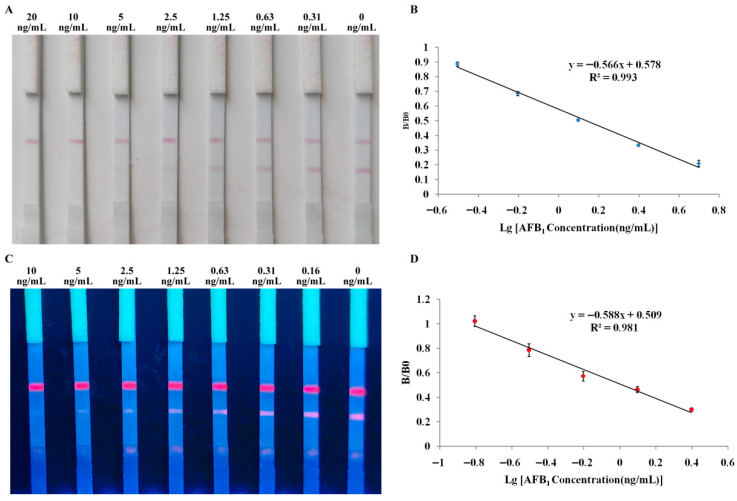
Sensitivity and standard curves of different detection modes. (**A**) Visible detection sensitivity of traditional colloidal gold LFIA; (**B**) Standard curve for traditional colloidal gold LFIA; (**C**) Visible detection sensitivity of traditional TRFN-LFIA; (**D**) Standard curve for traditional TRFN-LFIA.

Based on the traditional mode, the AFB_1_ colloidal gold and TRFM immunochromatographic assay were developed and used to evaluate the sensitivity of the novel TRFN-LFIA technology established in this study. As shown in Figure 6, the vLOD for both traditional colloidal gold-LFIA and TRFN-LFIA was 10 ng/mL, with IC_50_ values of 1.37 ng/mL and 1.04 ng/mL, respectively, and qLODs of 0.41 ng/mL and 0.32 ng/mL, respectively. From the results, it can be seen that, when the same raw materials and detection modes are used, the substitution of colloidal gold with TRFM can only slightly improve the sensitivity. Meanwhile, the novel TRFN-LFIA technology established in this study increased the quantitative sensitivity by approximately 10 times and the semi-quantitative sensitivity by 33 times.

## 4. Discussion

In the traditional LFIA mode, the labeling of antibodies with colloidal gold is a key factor affecting the performance of the test strips. During the labeling process, the number of labeled antibodies, their orientation, and spatial hindrance all significantly influence the detection performance. The ideal situation for labeled antibodies is that only one antibody is labeled on each colloidal gold to achieve optimal sensitivity. However, due to the complexity of the labeling process, precisely controlling the number of antibodies is almost impossible. Regarding the spatial hindrance, since the full length of an IgG molecule is only about 8 nm and only approximately 4 nm can extend from the surface of the gold particle [42], a high success rate in labeling can only be achieved when the coverage ratio of the antibody reaction determinants is less than 50% [43]. Therefore, it is difficult to precisely control the quantity and sites of labeled antibodies during the antibody labeling process, which affects the detection performance of the test strips. This study developed a novel LFIA mode that depends on several aspects: (A) Monoclonal antibodies do not require labeling, allowing for precise control of the antibody amount employed and minimizing steric hindrance, and the antigen in the sample preferentially reacts with mAbs, thereby enhancing detection sensitivity. (B) Using SPA as an alternative to artificial antigens for the test line, since each SPA molecule can bind to two IgG molecules, it can enhance interception efficiency, improving the sensitivity and accuracy of detection. (C) Select artificial antigens as labeled probes. Artificial antigens are formed by coupling carrier proteins with haptens, with each carrier protein carrying multiple hapten molecules, and each hapten possessing complete antigenic epitopes. This structural characteristic significantly reduces the impact of labeling position and spatial hindrance on antigen reactivity. Therefore, compared with labeled antibodies, labeled artificial antigens have more significant technical advantages.

Regarding the extraction coefficient, because the extraction efficiency during sample processing cannot achieve 100%, a deviation exists between the detection level and the spiked level. To account for this discrepancy, the extraction coefficient was derived as the ratio of the spiked level to the corresponding detection level in recovery experiments. This coefficient was subsequently applied to correct the raw detection data, ensuring that the recovery rates of all spiked samples fell within an acceptable range of 80–120%, thereby enhancing the accuracy and reliability of analytical results. In this study, an extraction coefficient of 1.85 was determined, indicating that the adopted extraction method achieves an AFB_1_ recovery rate of approximately 54.55%, which reflects moderate extraction efficiency and underscores the necessity for correction to ensure accurate quantification. In the established TRFN-LFIA, the minimum MF of the sample extraction solution is 2, and the extraction coefficient is 1.85. Based on this calculation, vLOD was found to be 0.30 ng/mL; with vLOD in samples being 2.22 µg/kg; qLOD was measured at 0.04 ng/mL, corresponding to qLOD in samples being 0.30 µg/kg.

To enhance the sensitivity of the LFIA for AFB_1_, researchers have explored various improvement strategies. Shim et al. [44] established an aptamer-based dipstick assay for detection of AFB_1_, with a vLOD of 10 ng/mL. Zhu et al. [28] established an up-converting phosphor technology-LFIA for rapid detection of AFB_1_ in feed, with LOD of 3 µg/kg. Tang et al. [45] established a TRFN-LFIA for the detection of AFB_1_ in maize based on Eu/Tb nanospheres and idiotypic nanobodies, with an IC_50_ of 0.46 ng/mL. Li et al. [46] established a TRFN-LFIA based on “green” extraction for the detection of AFB_1_ in corn, rice, and peanut, with LOD of 0.9 µg/kg. In comparison, the TRFN-LFIA developed in this study demonstrates superior sensitivity relative to these previously reported methods.

This study still has several limitations. To improve the extraction efficiency of the samples, high-concentration ethanol was used as the extraction solvent; however, its extraction efficiency was only 54.55%, which has become a key factor restricting the improvement of detection sensitivity. In addition, the LFIA exhibits limited tolerance to high-concentration ethanol, necessitating dilution of the sample extract solution prior to detection, which further compromises the method’s sensitivity. Therefore, developing a more efficient sample pretreatment method is expected to enhance the detection sensitivity of TRFN-LFIA.

## 5. Conclusions

In summary, a TRFN-LFIA for rapid and ultra-sensitive detection of AFB_1_ in food matrices was established. The entire testing process can be completed within 1 h. UV light irradiation enables visual detection of samples, rendering the method highly suitable for on-site rapid screening, with a visual detection limit (vLOD) of 0.30 ng/mL. A qLOD of 0.04 ng/mL was achieved via an immunofluorescence card reader; yielding an IC_50_ of 0.09 ng/mL and demonstrating a good linear correlation over the concentration range from 0.04 to 0.21 ng/mL. This method provides a rapid and accurate approach for the trace detection of AFB_1_ in food matrices and offers a novel strategy to enhance the sensitivity of LFIAs.

## Figures and Tables

**Table 1:** Cross-reactivity between AFB_1_ and its structural and functional analogs.

Analogue	IC_50_ (ng/mL)	CR (%)
AFB_1_	0.09	100%
AFB_2_	19.16	0.48
AFM_1_	2.54	3.58
AFG_1_	1.52	5.97
AFG_2_	3.96	2.30
ZEN	>10,000	<0.001
OTA	>10,000	<0.001
T2	>10,000	<0.001
DON	>10,000	<0.001

**Table 2:** Recovery of the TRFN-LFIA for the determination of AFB_1_ in spiked samples (*n* ≥ 3).

Sample	Spiked Level (µg/kg)	Detection Level (mean ± SD) (µg/kg)	Recovery (%)	CV (%)
Peanut	1	1.04 ± 0.14	103.88	13.29
	2	1.84 ± 0.13	91.81	6.99
	5	4.35 ± 0.40	86.91	9.10
	25	27.24 ± 2.75	108.97	10.09
Maize	1	0.81 ± 0.11	81.33	13.04
	2	2.36 ± 0.11	117.86	4.66
	5	5.52 ± 0.24	110.36	4.36
	25	24.65 ± 2.36	98.61	9.55

## Data Availability

The original contributions presented in this study are included in the article. Further inquiries can be directed to the corresponding author.
